# Optimizing evaluation of endometrial receptivity in recurrent pregnancy loss: a preliminary investigation integrating radiomics from multimodal ultrasound via machine learning

**DOI:** 10.3389/fendo.2024.1380829

**Published:** 2024-08-20

**Authors:** Shanling Yan, Fei Xiong, Yanfen Xin, Zhuyu Zhou, Wanqing Liu

**Affiliations:** ^1^ Department of Ultrasound, Deyang People’s Hospital, Deyang, Sichuan, China; ^2^ Department of Obstetrics and Gynecology, Deyang People’s Hospital, Deyang, Sichuan, China

**Keywords:** recurrent pregnancy loss, endometrial receptivity, radiomics, machine learning, shear wave elastography

## Abstract

**Background:**

Recurrent pregnancy loss (RPL) frequently links to a prolonged endometrial receptivity (ER) window, leading to the implantation of non-viable embryos. Existing ER assessment methods face challenges in reliability and invasiveness. Radiomics in medical imaging offers a non-invasive solution for ER analysis, but complex, non-linear radiomic-ER relationships in RPL require advanced analysis. Machine learning (ML) provides precision for interpreting these datasets, although research in integrating radiomics with ML for ER evaluation in RPL is limited.

**Objective:**

To develop and validate an ML model that employs radiomic features derived from multimodal transvaginal ultrasound images, focusing on improving ER evaluation in RPL.

**Methods:**

This retrospective, controlled study analyzed data from 346 unexplained RPL patients and 369 controls. The participants were divided into training and testing cohorts for model development and accuracy validation, respectively. Radiomic features derived from grayscale (GS) and shear wave elastography (SWE) images, obtained during the window of implantation, underwent a comprehensive five-step selection process. Five ML classifiers, each trained on either radiomic, clinical, or combined datasets, were trained for RPL risk stratification. The model demonstrating the highest performance in identifying RPL patients was selected for further validation using the testing cohort. The interpretability of this optimal model was augmented by applying Shapley additive explanations (SHAP) analysis.

**Results:**

Analysis of the training cohort (242 RPL, 258 controls) identified nine key radiomic features associated with RPL risk. The extreme gradient boosting (XGBoost) model, combining radiomic and clinical data, demonstrated superior discriminatory ability. This was evidenced by its area under the curve (AUC) score of 0.871, outperforming other ML classifiers. Validation in the testing cohort of 215 subjects (104 RPL, 111 controls) confirmed its accuracy (AUC: 0.844) and consistency. SHAP analysis identified four endometrial SWE features and two GS features, along with clinical variables like age, SAPI, and VI, as key determinants in RPL risk stratification.

**Conclusion:**

Integrating ML with radiomics from multimodal endometrial ultrasound during the WOI effectively identifies RPL patients. The XGBoost model, merging radiomic and clinical data, offers a non-invasive, accurate method for RPL management, significantly enhancing diagnosis and treatment.

## Introduction

In the quest to understand the complexities of recurrent pregnancy loss (RPL), a condition impacting up to 5% of couples striving for conception, a comprehensive exploration into its causes has been undertaken (1). This includes investigations into anatomical, endocrine, and immunological factors, among others (2). Despite these efforts, a significant proportion of RPL cases remain unexplained (3). It is recognized that the causes of RPL can generally be categorized into maternal and embryonic aspects (4). Recent studies have suggested that fetal chromosomal anomalies may account for 30 to 60% of miscarriages in RPL cases (5). It is noteworthy that chromosomal instability within preimplantation embryos is a common phenomenon, even among younger women of childbearing age (6). The maternal reproductive system is known to possess a natural quality control mechanism, designed to prevent the implantation of embryos with compromised viability (7). Thus, in many instances, RPL can be viewed as a failure of this natural selection process, resulting in the implantation and subsequent miscarriage of embryos unlikely to achieve full-term development.

Inadequate natural embryonic selection often results in a state of biological ‘superfertility’, characterized by insufficient decidualization of stromal cells and a misaligned maternal response to embryonic signals (8–10). This condition is thought to prolong the endometrial receptivity (ER) window, potentially leading to the delayed implantation of compromised embryos, a concept supported by research from Wilcox et al. (11). Since ER can be improved with individualized therapies (12), understanding the timed changes in the endometrial immune environment is key to assessing the optimal ER state, which could facilitate a balance between successful implantation and pregnancy in RPL women (13).

The clinical evaluation of the endometrium continues to be a critical component in the investigation of couples facing unexplained RPL (uRPL). Current research on ER predominantly focuses on endometrial parameters significant for predicting assisted reproduction outcomes, including endometrial morphology and Doppler blood flow assessed using ultrasonography (14, 15). However, the reliability of these parameters in identifying RPL patients remains a matter of debate (16). Invasive procedures like hysteroscopy, though offering detailed examination, are less suitable for routine screening and repeated measures (17). The progress in molecular testing offers hope, yet it requires extensive validation (18). Consequently, the development of more precise and objective non-invasive methods for ER assessment is essential for enhancing diagnostic accuracy in RPL and improving patient prognosis.

To address this need, our study introduces an enhanced approach by integrating radiomics into the established multimodal transvaginal ultrasound protocol. Radiomics, endorsed by the European Society of Radiology (19) as a leading-edge method in medical imaging, offers comprehensive feature extraction from imaging data (20), which facilitates potential clinical correlations in ER evaluation. Its recent application has shown promise in non-invasive ER evaluation (21). However, the complexity and non-linearity inherent in the relationships between radiomic features and clinical outcomes necessitate advanced analytical methods. Traditional linear models are inadequate for the required precision, highlighting the necessity for artificial intelligence, especially machine learning (ML) algorithms, to better analyze these intricate datasets (22). The combination of radiomics and ML presents a compelling synergy, particularly beneficial due to the large datasets provided by radiomics through its high-throughput extraction of quantitative features from medical images (23).

Given this potential, our study focuses on the development and validation of an ML model that utilizes radiomic features from grayscale (GS) and shear wave elastography (SWE) images of the endometrium obtained via transvaginal ultrasound. The aim is to refine ER evaluation in RPL patients, facilitating the identification of specific ER states. This improved identification process is crucial for the timely application of customized therapies, addressing the unique needs of RPL patients.

## Materials and methods

Conducted with a retrospective and controlled methodology, this study adhered rigorously to the ethical guidelines outlined in the Declaration of Helsinki. Ethical approval was secured from the Institutional Review Board of Deyang People’s Hospital (2022-04-083-K01). In light of the study’s retrospective nature, the requirement for informed consent was waived by the ethical committee. To ensure the confidentiality and privacy of the participants, a comprehensive anonymization process was applied to all participant data before their inclusion in the research analysis.

### Subjects

Between 2021 and 2023, data from 400 patients with uRPL were collected for the RPL group. These cases were defined as experiencing the consecutive spontaneous loss of two or more clinically recognized pregnancies before the 24th week of gestation, based on the criteria from the European Society of Human Reproduction and Embryology (ESHRE) and the American Society for Reproductive Medicine (ASRM) (24, 25). This definition excludes ectopic, molar, and biochemical pregnancies. Autoimmune, anatomic, genetic, endocrine, infectious, and male factors were excluded upon initial assessment. For the control group, 400 women seeking to enhance their chances of conception were selected. These control subjects had undergone various assessments at our center, including evaluations of ovarian reserve and ER, and had subsequently achieved a full-term pregnancy without previous pregnancy loss.

Criteria for inclusion of both groups encompassed an age range of 20 to 40 years, regular menstrual cycles of 27 to 35 days, and normal ovarian reserve. Participants were also required to have normal ovarian and uterine ultrasonography, absent of cysts, fibroids, polyps, or significant structural anomalies, and a history free from major gynecological surgeries, except minor procedures like curettage, diagnostic laparoscopy, and hysteroscopy. Women with a history of heavy drinking, systemic diseases affecting hemodynamic indexes, or recent use of steroid hormones, antibiotics, or other medications influencing pregnancy outcomes, were excluded from both groups.

Following rigorous selection processes, 346 RPL patients and 369 control individuals were enrolled in this study. To ensure the robustness and validity of our model, the subjects were randomly assigned to a training cohort of 500 individuals (242 RPL, 258 controls) and a validation cohort of 215 individuals (104 RPL, 111 controls) in a 7:3 ratio. Comprehensive clinical data collected during the initial consultation included age, body mass index (BMI), history of previous miscarriages, and ovarian reserve indicators such as follicle-stimulating hormone (FSH), luteinizing hormone (LH), estradiol (E_2_), antral follicular count (AFC), and antimüllerian hormone (AMH) levels.

### Transvaginal ultrasound for ER

During the window of implantation (WOI), typically 7-9 days following ovulation (days 21–23 of the cycle), uniform transvaginal ultrasound scanning was performed on all subjects using the Resona R9T system (Shenzhen Mindray Corporation, Shenzhen, China). The standard measurements included endometrial thickness (EMT), as well as the analysis of blood flow dynamics within the uterine arteries (UA) and the spiral arteries (SA). This analysis incorporated the calculation of the mean pulsatility index (PI) and resistance index (RI) for the bilateral UAs and SAs. Additionally, SWE and three-dimensional (3D) imaging modes were routinely employed as part of the ER assessment. Following the manual delineation of the endometrial outline, the system autonomously calculated various parameters including the Young’s modulus value of the endometrium and volumetric data. This data encompassed the endometrial volume, along with the vascularization index (VI), flow index (FI), and vascularization flow index (VFI). The VI was defined as the proportion of power Doppler information, the FI reflected the power Doppler signal’s intensity, and the VFI integrated both these measurements (26). To enhance the reliability of these assessments, each examination was repeated twice and the average values were recorded.

### Endometrial segmentation process

**Figure 1:**
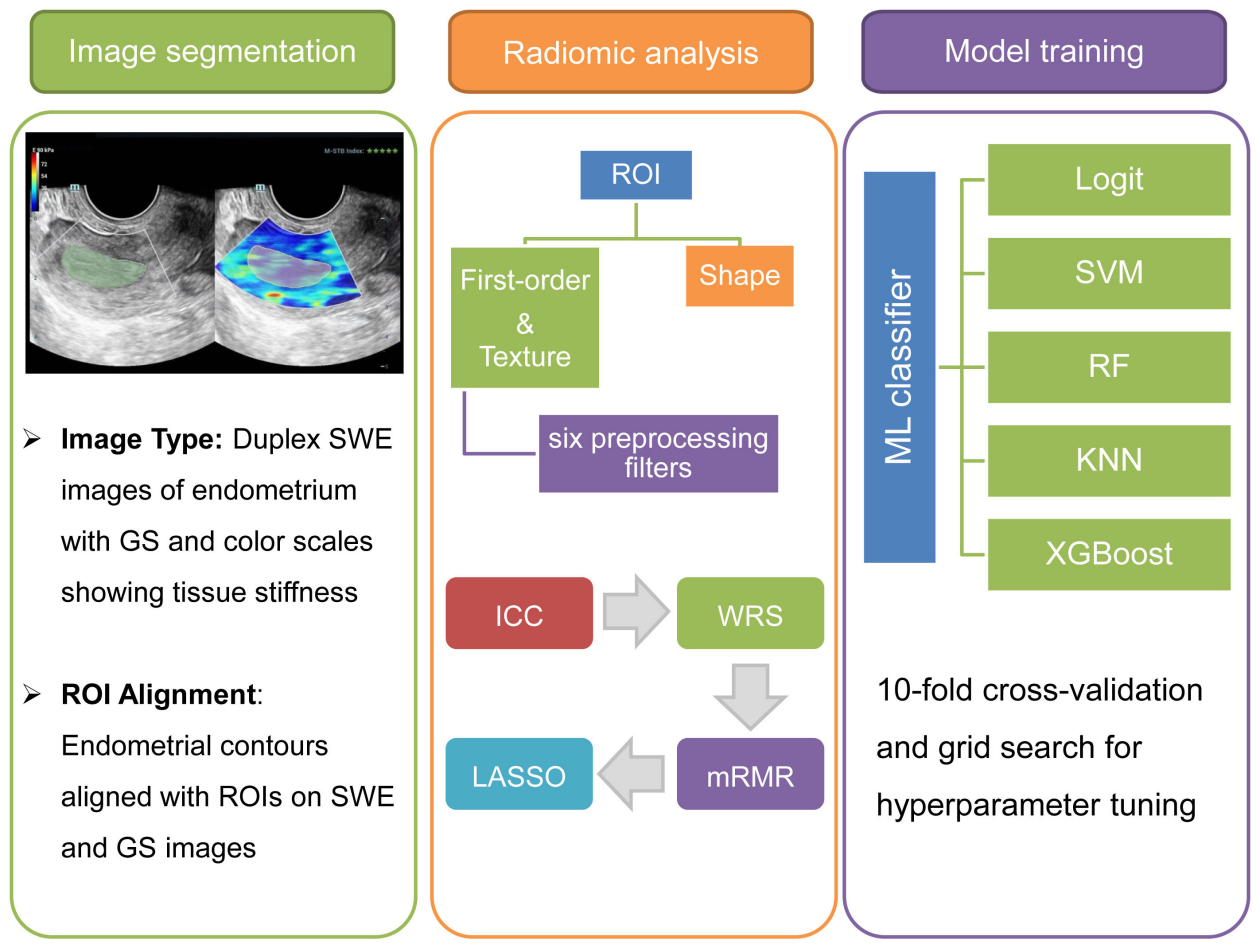
Multimodal radiomics-based ML workflow for ER assessment. The depicted workflow begins with endometrial segmentation through Duplex SWE imaging. It advances to comprehensive radiomic analysis and concludes with the refinement and optimization of multiple ML classifiers for precise assessment.

Endometrial segmentation was performed on offline Duplex SWE images, which depicted the endometrium in a longitudinal section. These images featured a dual representation of GS and SWE color scales, reflecting tissue stiffness variations from lower (deep blue) to higher (red) levels. As outlined in **Figure 1**, the workflow involved sequential stages of image segmentation, radiomic analysis, and the training of ML models. Segmentation was executed using the 3D Slicer software (version 5.6.1), focusing on the precise delineation of the entire endometrium as the region of interest (ROI). Two expert sonographers, unaware of the study’s objectives, meticulously marked these ROIs to ensure observer consistency. ROIs on the right side of the SWE images were aligned with the endometrial contours, while corresponding ROIs were identified on the left-side GS images.

### Radiomic feature extraction

Subsequent to the delineation of ROIs, the Pyradiomics toolkit was employed for extracting radiomic features from the segmented images. This step transformed segmented medical images into a highly structured dataset with multidimensional attributes, crucial for the quantification and characterization of ER. A total of 1316 unique features were extracted from each segmented image, comprising 12 shape-related, 18 first-order statistical, and 75 textural features from the initial images. The textural features were further categorized based on their originating matrix, including gray-level co-occurrence, gray-level dependence, gray-level run length, gray-level size zone, and neighboring gray tone difference. In addition, six preprocessing filters (Exponential, Gradient, Logarithm, Square, Square-root, and Wavelet) were applied to the initial images, generating an additional 1209 filtered features. All extracted features were systematically cataloged in an Excel file for the subsequent feature selection process.

### Radiomic and clinical data preprocessing

In preparation for the predictive model development, a critical data preprocessing step, encompassing both extracted radiomic features and clinical data, was undertaken to normalize the comprehensive dataset, thereby ensuring the integrity and objectivity of the subsequent analysis. Continuous variables were normalized using the Z-score method, aligning them to a standard scale with a mean of zero and a standard deviation of one. Meanwhile, categorical variables underwent binary transformation, being encoded as ‘0’ and ‘1’. In defining clinical outcomes, patients with RPL were coded as ‘1’, distinguishing them from control subjects, who were coded as ‘0’.

### Radiomic feature selection

For the radiomic features extracted from each segmented image, a structured multi-step process was utilized to select features associated with RPL. This process initiated with the assessment of interobserver agreement, quantified using the intraclass correlation coefficient (ICC) with a threshold of 0.8 to ensure observer concordance. Subsequent statistical analyses began with the Wilcoxon rank sum (WRS) test, identifying RPL-related features based on a false discovery rate-adjusted P-value under 0.1. Further refinement employed the minimum redundancy maximum relevance (mRMR) method, isolating the top 20 features with high relevance and minimal redundancy to RPL. The final selection phase applied least absolute shrinkage and selection operator (LASSO) logistic regression, focusing on isolating the most predictive features for RPL.

### Training of ML models

The entire model training process, from algorithm selection to hyperparameter tuning, was executed using the Scikit-Learn library in Python. Five supervised ML classifiers were deployed for RPL risk stratification. These classifiers included logistic regression (Logit), support vector machines (SVM), random forests (RF), k-nearest neighbors (KNN), and extreme gradient boosting (XGBoost). Hyperparameter optimization was conducted using a grid search algorithm, detailed in **Supplementary Table S1**, to mitigate overfitting and enhance model robustness. For data partitioning, a 10-fold cross-validation method was adopted. This involved sequentially segmenting the dataset into ten subsets, using each in turn as an inner validation set while the remaining subsets constituted the training set.

### Internal and external validations of ML models

For each participant in the training cohort, three distinct sets of ML models were developed based on radiomic data, clinical data, and a combined dataset of both. These models underwent a thorough internal validation process to assess their discriminative accuracy, calibration, and clinical applicability. The selection of the most effective model was informed by its excellence in discrimination, robust calibration, and relevance in a clinical setting. External validation of the optimal model was conducted using the testing cohort, also focusing on assessing the model’s discrimination, calibration, and clinical utility, thereby ensuring its clinical applicability. To comprehensively quantify the model’s utility in practical scenarios, key performance metrics, including accuracy, precision, recall, and F1 score, were analyzed within the testing cohort.

### Interpretability of the optimal ML model

In order to demystify the inherent opacity of ML models, we employed the Shapley Additive Explanations (SHAP) approach. This technique quantifies and ranks the influence of each variable on the model’s predictions, offering a clear depiction of their relative importance (27). By arranging features in descending order based on SHAP values, the key predictive factors within the model are highlighted. To further assess potential collinearity among the most influential variables, we generated a heatmap of the correlation matrix for these top predictors. This visualization helps identify any redundancy or high collinearity that could affect the model’s quality.

### Statistical analysis

A tailored approach to statistical analysis was employed to discern differences between training and testing cohorts. Continuous data, depending on their distribution, were subjected either to the independent-sample t-test (for normally distributed data) or the Mann–Whitney U test (for non-normally distributed data). Model performance was evaluated through receiver operating characteristic (ROC) curve analysis, with the area under the curve (AUC) assessing discrimination capability. AUC values were compared using Delong’s test. The goodness of fit for each model was assessed using calibration curve analysis and the Brier Score. To determine the clinical applicability, decision curve analysis (DCA) was implemented for evaluating net benefits at varied threshold probabilities. All analyses were performed using Python (version 3.12.0), considering a p-value below 0.05 as statistically significant.

## Results

### Cohort characteristics

**Figure 2:**
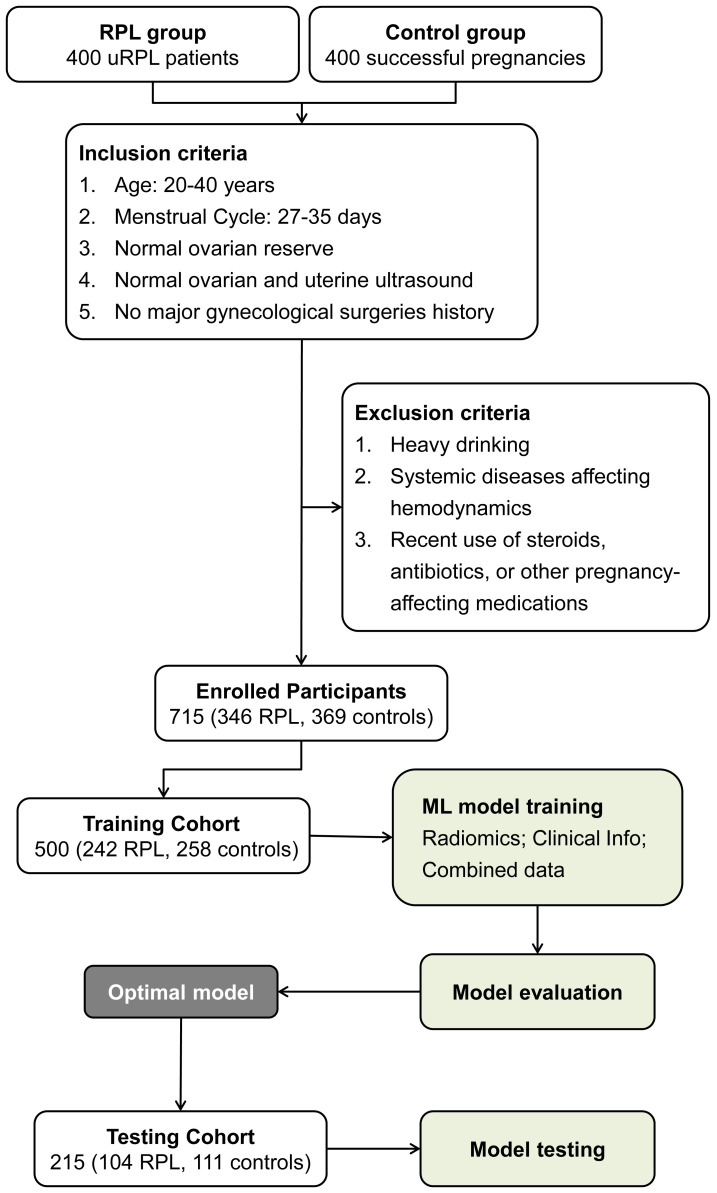
Workflow illustrating participant selection and cohort distribution for ML model development in RPL risk assessment.

**Figure 2** illustrates the workflow for participant selection and the subsequent development phases of the ML model in this study. A total of 715 participants were enrolled, comprising 346 individuals with RPL and 369 controls. Within the RPL sufferers, recurrent miscarriage occurrences were 58.3% for two, 30.6% for three, and 10.9% for four or more. These participants were randomly assigned to training and testing cohorts for model development and validation. **Table 1** reveals uniform demographic and clinical features across both cohorts, with no significant disparities in baseline characteristics (all P-values > 0.05), ensuring a balanced evaluative basis.

**Table 1:** Comparative analysis of demographic and clinical parameters between training and testing cohorts.

Indicators	Training cohort (n = 500)	Testing cohort (n = 215)	*t/z* value	*P* value
Age, year	33 (30–35)	33 (31–35)	1.011	0.312
BMI, kg/m^2^	21.52 ± 3.41	21.71 ± 3.79	0.654	0.513
FSH, IU/L	7.27 (6.17-8.69)	7.19 (6.01-8.63)	0.559	0.576
LH, IU/L	6.75 (5.89-7.65)	6.75 (5.89-7.58)	0.080	0.937
E_2_, pg/mL	34.9 (29.6-39.3)	34.2 (29.2-39.7)	0.073	0.941
AMH, ng/ml	1.47 ± 0.42	1.49 ± 0.41	0.566	0.571
EMT, mm	8.91 ± 1.79	8.88 ± 1.87	0.230	0.818
Endometrial volume, ml	4.95 ± 0.96	4.95 ± 0.98	0.060	0.952
Young’s modulus value, kPa	12.5 (10.6-14.3)	12.7 (10.9-14.3)	0.988	0.323
SAPI	0.97 (0.85-1.13)	0.96 (0.84-1.12)	0.475	0.635
SARI	0.54 ± 0.05	0.54 ± 0.52	0.723	0.470
UAPI	2.12 ± 0.20	2.12 ± 0.21	0.092	0.927
UARI	0.82 (0.80-0.84)	0.83 (0.79-0.84)	0.085	0.932
VI, %	2.39 ± 0.59	2.44 ± 0.58	1.062	0.289
FI	26.78 ± 6.58	27.39 ± 6.42	1.135	0.257
VFI	0.60 (0.47-0.78)	0.60 (0.48-0.78)	0.472	0.637

### Radiomic features extraction and selection

**Figure 3:**
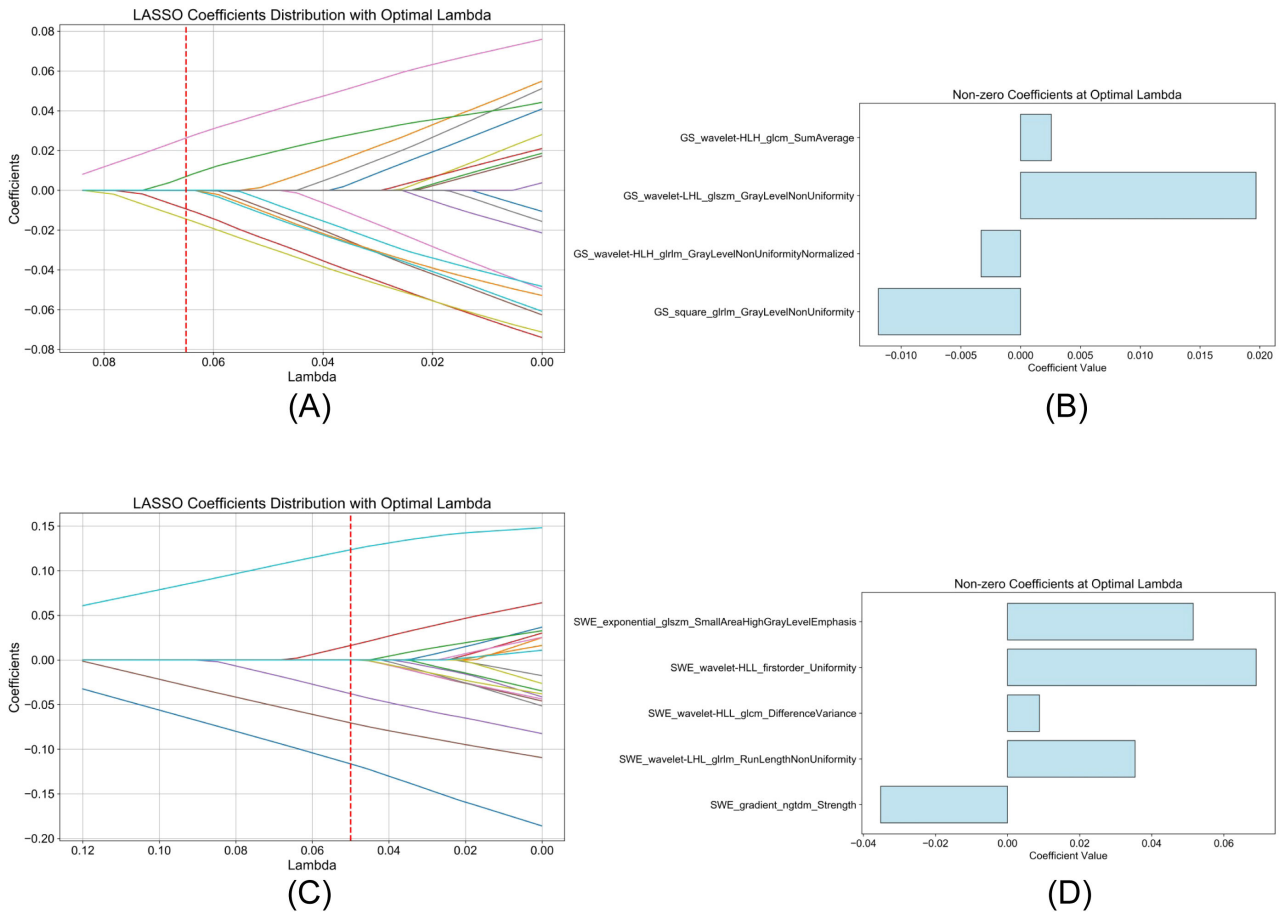
LASSO logistic regression analysis of radiomic features. **(A, C)** display the trajectory of LASSO coefficients for GS and SWE features, respectively, with the optimal lambda value indicated by the vertical red dashed line. **(B, D)** highlight the selected features with non-zero coefficients at this optimal lambda value, demonstrating their significance in the assessment of RPL risk.

In the training cohort of this study, the delineation of endometrial ROIs in GS and SWE modalities was conducted on duplex transvaginal ultrasound images for each participant. This process led to the extraction of a comprehensive set of 2626 radiomic features, with an equal distribution across both GS and SWE images. Subsequent standardization procedures resulted in the identification of 1145 GS-derived features and 1202 SWE-derived features, each demonstrating an ICC equal to or above 0.8, thereby qualifying for further analysis. The WRS test identified 117 GS and 141 SWE features as potential indicators of increased RPL risk. Subsequent refinement via the mRMR algorithm shortlisted the top 20 features from each modality, prioritizing those with maximal relevance to RPL risk and minimal redundancy. The final phase of feature selection involved LASSO logistic regression, which highlighted 4 GS and 5 SWE features with significant RPL risk correlations, each exhibiting non-zero coefficients. The distribution patterns of these selected features are illustrated in **Figure 3**, along with detailed descriptions and weight information in **Supplementary Table S2**.

### Training and evaluation of ML models

**Figure 4:**
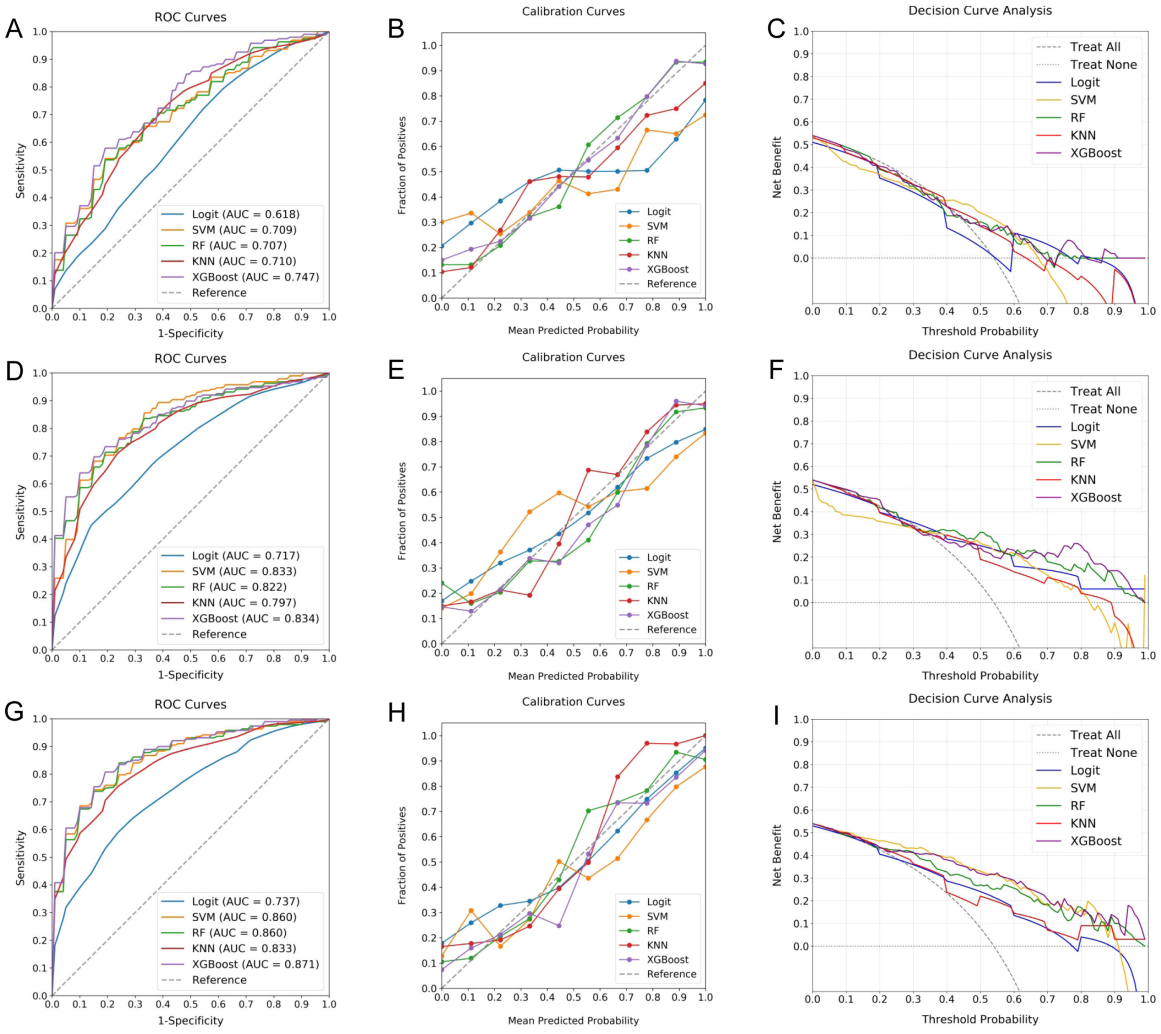
Comparative performance of ML classifiers for RPL risk assessment. **(A–C)** evaluate models using clinical data, **(D–F)** focus on radiomic features, and **(G–I)** combine both datasets, depicted through ROC curves, calibration plots, and DCA. These visualizations provide insights into the discriminative accuracy, calibration, and clinical utility of the Logit, SVM, RF, KNN, and XGBoost classifiers, with RF and XGBoost showing superior performance, particularly when leveraging the integrated dataset.

Employing the nine selected radiomic features and standardized clinical data, the efficacy of five distinct ML classifiers was explored. These included Logit, SVM, RF, KNN, and XGBoost. Each classifier underwent a comprehensive optimization process facilitated by 10-fold cross-validation, ensuring hyperparameter refinement for optimal performance. **Figure 4** presents a comparative analysis of these models, organized into three sets. The outcomes from models based on radiomic features are shown in sections **Figures 4A–C**, those based on clinical data in **Figures 4D–F**, and models utilizing a combination of both in **Figures 4G–I**. Each section includes ROC curves, calibration plots, and DCA, providing a multifaceted view of each classifier’s predictive capacity in the context of elevated RPL risk.

The investigation found that models integrating both clinical and radiomic data yielded superior discriminative ability, as reflected in the AUC values. Models combining both types of data exhibited AUCs ranging from 0.737 to 0.871, outperforming those based solely on clinical (0.618 to 0.747) or radiomic features (0.717 to 0.834). Notably, the RF and XGBoost classifiers achieved the highest AUCs of 0.860 and 0.871, respectively. These models also showed impressive calibration, characterized by calibration curves closely approximating the 45-degree line and low Brier Scores (RF: 0.0052, XGBoost: 0.0062), thereby enhancing their predictive reliability. Furthermore, both RF and XGBoost demonstrated significant clinical utility, offering substantial net benefits across a broad threshold probability range (20-100%). Despite their close performance, XGBoost marginally outperformed RF, emerging as the optimal choice for assessing RPL risk.

### Validation of the optimal model

**Figure 5:**
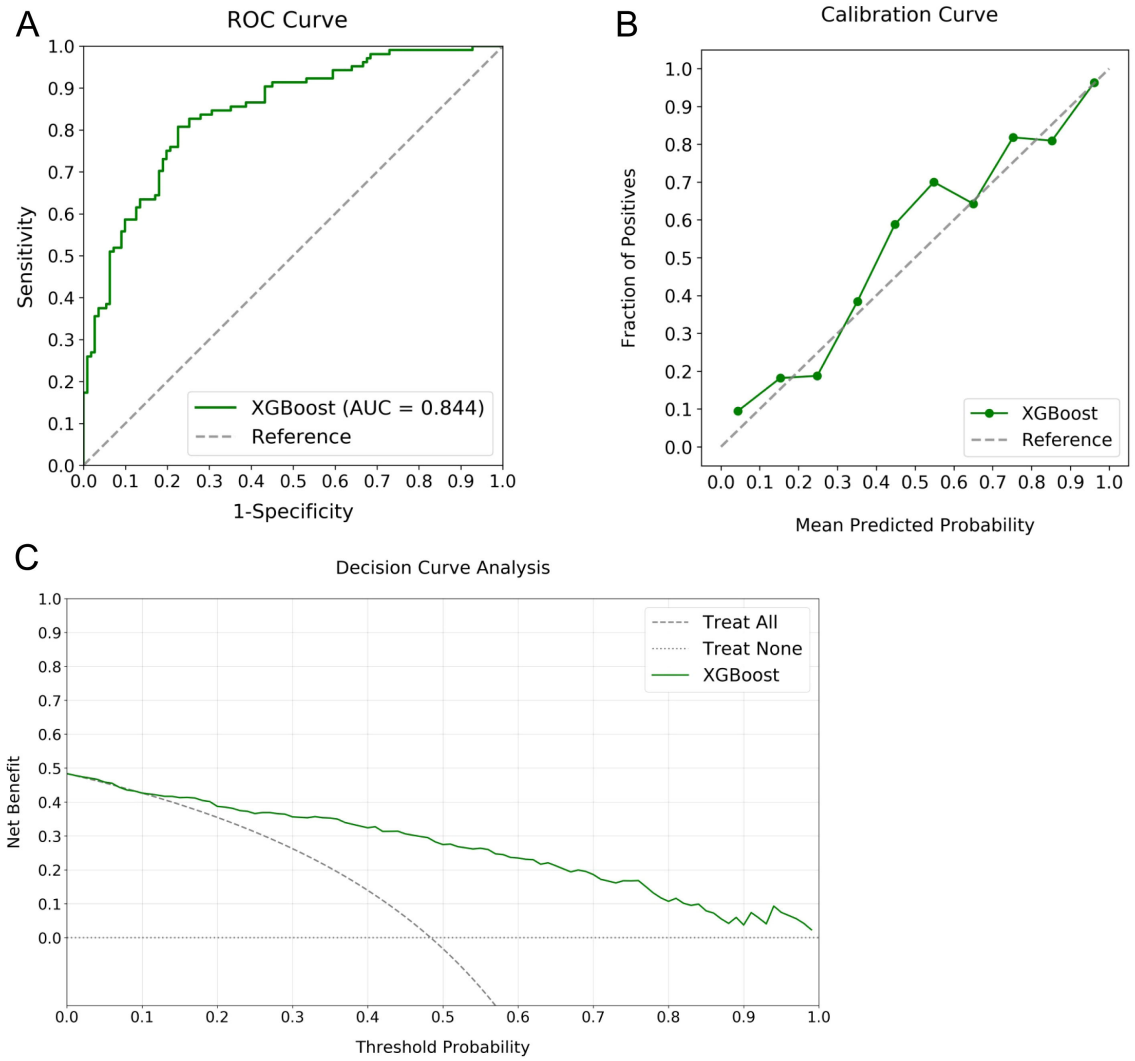
Validation metrics for the XGBoost model in RPL risk stratification. Panel **(A)** displays the ROC curve with an AUC of 0.844, panel **(B)** shows the calibration curve illustrating agreement between predicted probabilities and observed frequencies, and panel **(C)** depicts the DCA indicating the notable net benefits at different threshold probabilities.

The robustness of the XGBoost model for RPL risk stratification was validated using an independent testing cohort composed of RPL and control women, which was not used in the training phase, ensuring an unbiased evaluation of the model’s performance. Analysis involved inputting cohort data into the model, yielding estimations subsequently compared with the actual status of participants. **Figure 5** details the validation outcomes, featuring an ROC curve with an AUC of 0.844, indicating substantial predictive accuracy (**Figure 5A**). The calibration curve reflected close agreement between the predicted probabilities and the observed frequencies, demonstrating model calibration integrity (**Figure 5B**). DCA revealed significant clinical net benefit for probability thresholds exceeding 10% (**Figure 5C**). Performance indicators derived from the confusion matrix, including accuracy, precision, recall, and F1 score, were determined to be 0.803, 0.850, 0.704, and 0.770, respectively. These statistics reinforce the XGBoost model as a robust tool for RPL risk evaluation.

### Model interpretation

**Figure 6:**
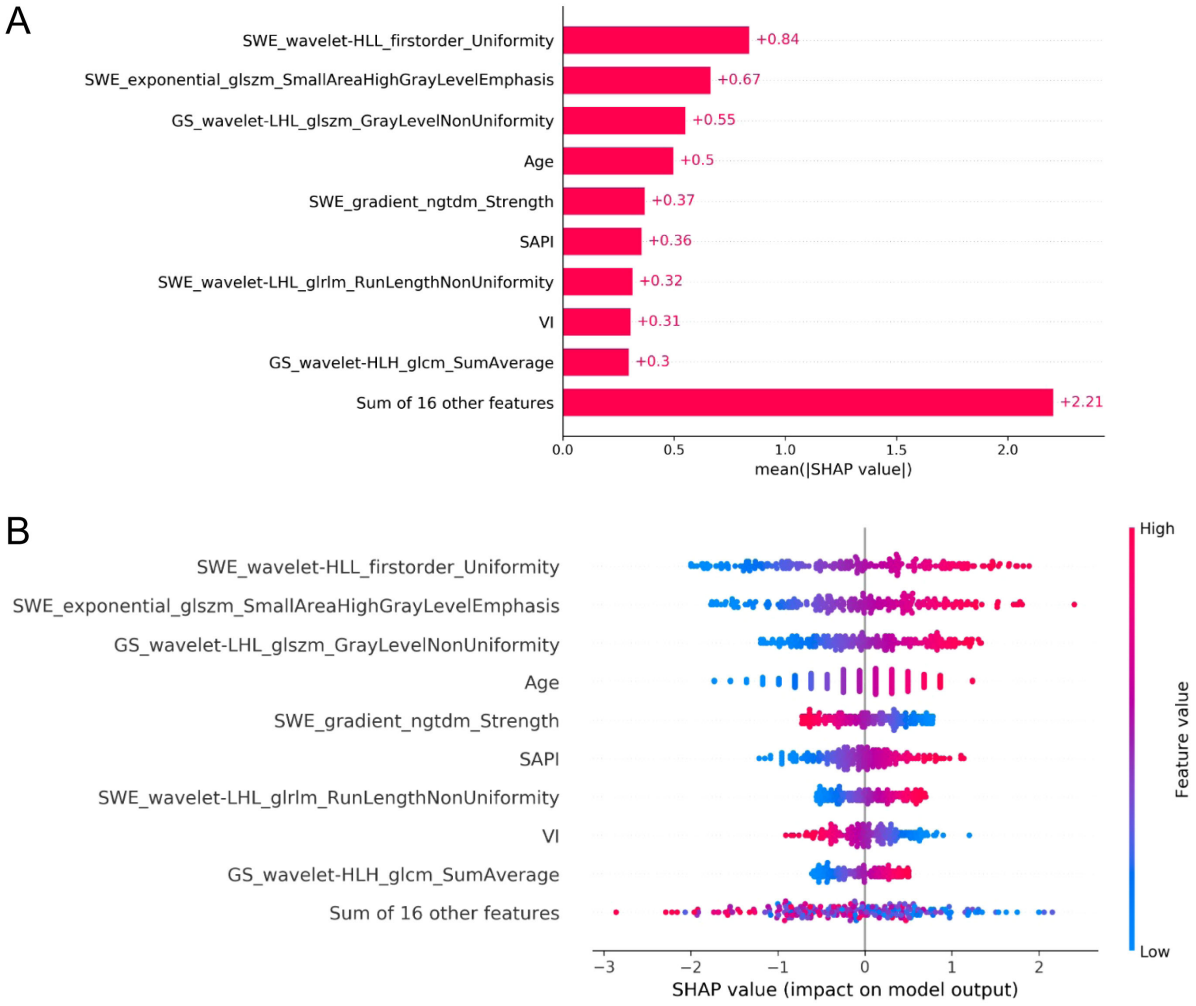
SHAP analysis for feature importance in the XGBoost model for RPL risk evaluation. **(A)** ranks the predictors by mean absolute SHAP values, highlighting the most impactful radiomic features from SWE and GS endometrial imaging, along with key clinical variables. **(B)** provides a summary plot illustrating the aggregate effect of these predictors on RPL risk, with the color gradient from blue to red denoting increasing feature values. The horizontal placement of data points represents the impact of SHAP values on risk prediction, with rightward and leftward points suggesting higher and lower RPL risk, respectively.

**Figure 7:**
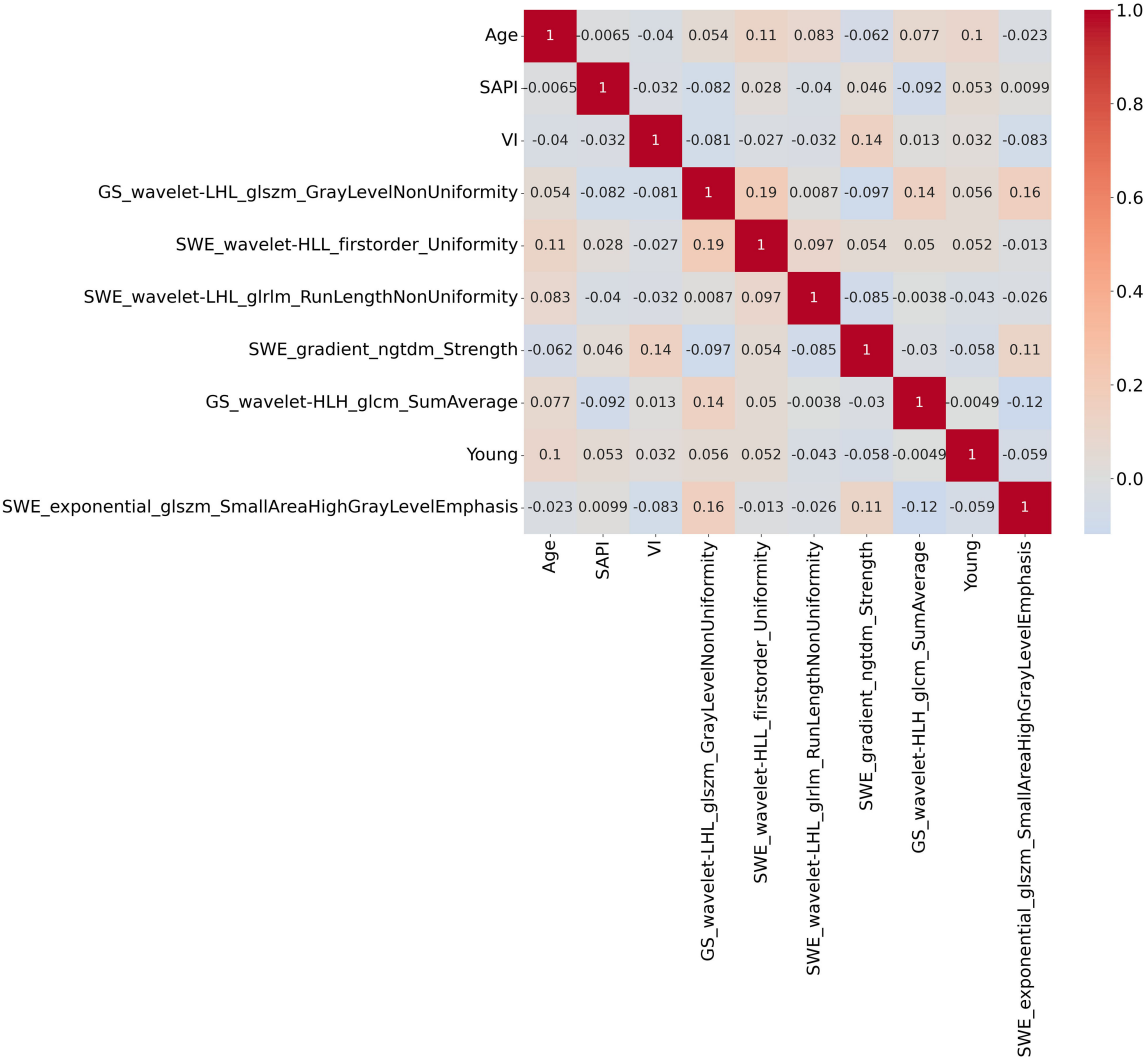
Heatmap of the correlation matrix for the nine critical features identified by SHAP analysis. The heatmap demonstrates minimal inter-feature correlation, with the highest correlation coefficient being less than 0.2. This indicates low collinearity among the features, suggesting that each feature independently contributes to the prediction accuracy of RPL risk.

SHAP value analysis enhanced the interpretability of the XGBoost model for RPL risk assessment by quantifying the contribution of each predictor. Mean absolute SHAP values identified four significant radiomic features from endometrial SWE images, two from GS images, and clinical variables such as age, SAPI, and VI as key determinants. As shown in **Figure 6A**, these features are ranked by their SHAP values, with **Figure 6B** providing a detailed visualization of their combined effects. The top four indicators, each with a mean impact exceeding 0.5, include two radiomic features from SWE images, one from GS images, and age. **Figure 7** presents a heatmap of the correlation matrix for these nine critical features, showing minimal inter-feature correlation, with the highest correlation coefficient being less than 0.2. This indicates low collinearity, confirming that each feature independently contributes to the prediction accuracy for RPL risk.

## Discussion

In the specialized field of RPL, this study innovates by incorporating ML techniques to interpret complex radiomic data from transvaginal ultrasound. Focused on enhancing ER assessment for RPL risk stratification, it integrates quantitative radiomic features from both GS and SWE images of the endometrium. Crucial to this approach is the finding that the XGBoost model excels as the most effective tool. This model, relying on a selected group of 4 GS and 5 SWE features, along with key clinical parameters including age, SAPI, and VI, effectively identifies distinct ER patterns. The robustness of the XGBoost model is consistently demonstrated across both training and validation cohorts, affirming its reliability and accuracy. This method offers a non-invasive, reproducible way to differentiate RPL patients from healthy individuals, potentially guiding more targeted and effective treatments.

Accurate ER evaluation is crucial for identifying RPL risk. There is a clear connection between RPL and the disrupted process of decidualization, where endometrial stromal cells transform into decidual cells (28). This key transformation concludes the implantation window and enables the endometrium to identify, react to, and remove non-viable embryos (29). Impairments in this functional aspect of decidualization increase the likelihood associated with delayed implantation, insufficient embryo quality control, and early placental dysfunction (30). Moreover, enhancing ER through personalized treatments highlights the need for optimal ER state assessment (31). Such assessments are crucial not only for identifying women at risk of RPL but also for improving their endometrial conditions, thus potentially boosting their pregnancy success rates.

The utilization of radiomics, characterized by a range of mathematically extracted parameters, has attracted considerable attention due to its potential in delineating heterogeneity within specific regions (32). In reproductive medicine, these parameters are promising for advancing clinical diagnostics and prognostication, providing a non-invasive method to detect subtle microstructural details, which surpasses the capabilities of conventional ultrasonography (33). The introduction of radiomics in identifying features associated with RPL represents a significant advancement toward innovative therapeutic interventions and preventive strategies. By facilitating detailed assessment of ER, radiomics enables clinicians to customize interventions to enhance the uterine environment for pregnancy. In this context, the study conducted by Huang et al. (21) represents a pioneering exploration, identifying unique radiomic characteristics associated with uRPL and showing advantages over traditional ER indicators. However, these findings await further validation in test cohorts for the assessment of their predictive robustness and stability.

Our study extends previous research by extracting radiomic features from both GS and SWE ultrasound imaging of the endometrium, thereby broadening the scope of endometrial condition evaluation. The extraction of approximately 2600 radiomic features from both GS and SWE endometrial segmentation in each subject, and the subsequent analysis of over one million data points in the training cohort of 500 participants, underscores the complexity and high-dimensional nature of this dataset. Such intricacy and the interplay of multiple factors render traditional linear predictive models inadequate, necessitating the application of ML algorithms (34). After a series of optimizations and comparative evaluations, it was observed that models combining both radiomic and clinical indicators outperformed those based solely on either type of data. Among the various ML models, the XGBoost algorithm emerged as the most effective in stratifying RPL risk, demonstrating high and consistent predictive performance in the testing cohort. This underlines the ability of the XGBoost model to proficiently manage large datasets and its robustness against overfitting, efficiently handling non-linearities and interactions between features, making it particularly suitable for complex datasets (29).

Employing SHAP for feature significance evaluation and a heatmap to assess collinearity, this investigation quantified individual feature impacts on model predictions, thereby enhancing model interpretability (35). The integration of SHAP with the XGBoost model rendered a transparent illustration of the paramount impact of nine critical variables, highlighting four SWE and two GS radiomic indices. This suggests a greater relevance of SWE attributes, which represent endometrial stiffness, in RPL compared to GS indicators, yet the Young’s modulus value, indicative of mean endometrial elasticity, did not emerge as a highly correlated variable with RPL. The identified SWE and GS radiomic features predominantly encompassed first-order and textural characteristics, reflective of image heterogeneity and high-intensity regions. These features, challenging to identify through traditional visual analysis, necessitated the application of multiple filters for quantifying the uniformity, variability, and anomalies within endometrial imagery, thereby establishing their correlation with RPL.

Meanwhile, SHAP analysis revealed the non-negligible contributions of age, SAPI, and VI to the stratification of RPL risk. These clinical parameters, not derivable from ultrasonic radiomics, have demonstrated associations with RPL in previous reports (36–38). It is acknowledged that RPL women often exhibit an extended WOI, leading to diminished endometrial perfusion during the implantation window, typically observed 7-9 days post-ovulation (39). This impaired perfusion, characterized by heightened vascular resistance and suboptimal blood flow distribution (34), was evident in our findings through increased SAPI and lower VI compared to controls. Additionally, it is important to note that with advancing age, a more pronounced increase in the likelihood of these aberrations is observed (40). Hence, the integration of clinical variables remains crucial for a comprehensive and accurate prediction. Despite the complexity in interpreting the linkage between radiomic features and RPL, the potential of radiomics in predictive analysis is evident. These features could facilitate more precise and extensive assessments of ER, potentially filling existing gaps in the understanding of the etiology of uRPL. By extracting detailed information about the endometrium, radiomics could enrich our comprehension of ER, thereby aiding in refining diagnostic and therapeutic strategies for RPL. This includes leveraging the model’s predictive capabilities to guide clinical interventions, such as adjusting hormonal therapy and optimizing the timing of embryo transfer, based on the identified ER states, ultimately improving pregnancy success rates.

In the preliminary investigation of integrating radiomics and ML with multimodal transvaginal ultrasound for stratifying RPL, the findings are promising but constrained by several factors. The single-center design and limited cohort size may not reflect the broader population accurately. Additionally, variability in GS and SWE settings across institutions could impact the reproducibility and effectiveness of the proposed models. The retrospective nature of the study limited it to recording the number of miscarriages at the initial visit, thus not exploring the correlation between miscarriage frequency and RPL risk, and precluded the prediction of subsequent pregnancy success rates in RPL patients. Although the inclusion and exclusion criteria effectively screened out many conditions that play a crucial role in implantation, such as polycystic ovary syndrome and endometriosis, the study design also limited the use of more advanced and precise detection techniques, potentially leaving some cases undetected.

Considering the potential for intervention and modification of endometrial receptivity (ER), irrespective of RPL status, future research should focus on larger, multicenter prospective studies. These studies are necessary not only to validate and refine the ML models but also to improve the prediction of successful ER regulation by incorporating more advanced detection techniques, such as the assessment of immunological interactions between the embryo and the endometrium. This approach will lead to better outcomes for RPL patients.

In conclusion, this research demonstrates the effectiveness of the XGBoost model in accurately identifying RPL patients. Utilizing GS and SWE radiomic features derived from duplex ultrasonography of endometrium, coupled with clinical factors such as age, SAPI, and VI, robust results were observed in both training and validation cohorts. This integration of radiomics-based ML represents a significant advancement in precision medicine, offering a more refined approach to RPL risk stratification. Such enhanced accuracy and predictive capacity of the model show promise in facilitating more individualized management of RPL.

## Data Availability

The raw data supporting the conclusions of this article will be made available by the authors, without undue reservation.
