# Supplementary material for: Optimizing evaluation of endometrial receptivity in recurrent pregnancy loss: a preliminary investigation integrating radiomics from multimodal ultrasound via machine learning
Source: Front Endocrinol (Lausanne). 2024 Aug 20;15:1380829. doi: 10.3389/fendo.2024.1380829 (PMC11368730; doi:10.3389/fendo.2024.1380829)
Supplement: Supplementary file 1 [file Table1.docx]

**Supplementary**

**Table S1**. Hyperparameter tuning grid for ML models

| Model | Hyperparameter | Searched value |
| --- | --- | --- |
| Logit | Regularization Strength | 1e-1, 1, 10 |
|  | Penalty | Ridge regularization ('l2') |
| SVM | Regularization Parameter | 1e-2，1e-1, 1, 10 |
|  | Kernel | 'linear', 'rbf', 'poly' |
|  | Tolerance | 1e-5, 1e-4, 1e-3 |
| RF | Number of Trees | 100, 300, 500 |
|  | Maximum Depth of Tree | 4, 6, 8 |
|  | Minimum Samples for Split | 2, 5, 10 |
| KNN | Number of Neighbors | 3, 5, 7, 9 |
|  | Weight Function | 'uniform', 'distance' |
|  | Distance Metric | 'euclidean','manhattan', 'minkowski' |
| XGBoost | Number of Boosting Rounds | 100, 200,300 |
|  | Learning Rate | 0.01, 0.1, 0.2 |
|  | Maximum Depth of Trees | 3, 4, 5 |

**Table S2**. Characteristics and weights of radiomic features selected by LASSO logistic regression

| Position | Filter | Name | Description | Weight |
| --- | --- | --- | --- | --- |
| GS radiomics | wavelet-HLH | glcm_SumAverage | Reflects the average sum of gray-level values in the image texture. | 0.001 |
|  | wavelet-LHL | glszm_GrayLevelNonUniformity | Measures the variability of gray-level intensity zones. | 0.020 |
|  | wavelet-HLH | glrlm_GrayLevelNonUniformityNormalized | Indicates normalized texture non-uniformity. | -0.003 |
|  | square | glrlm_GrayLevelNonUniformity | Evaluates the non-uniformity of gray levels in texture. | -0.012 |
| SWE radiomics | exponential | glszm_SmallAreaHighGrayLevelEmphasis | Focuses on small zones with high intensity. | 0.051 |
|  | wavelet-HLL | firstorder_Uniformity | Assesses the uniformity of gray-level values | 0.069 |
|  | wavelet-HLL | glcm_DifferenceVariance | Measures the variance of gray-level differences. | 0.009 |
|  | wavelet-LHL | glrlm_RunLengthNonUniformity | Reflects the non-uniformity of run lengths in texture. | 0.035 |
|  | gradient | ngtdm_Strength | Indicates the contrast strength in texture patterns. | -0.035 |
